# National Dental Association, Omaha, Nebraska, 1898

**Published:** 1898-11

**Authors:** Jennie M. Walker

**Affiliations:** Bays St. Louis, Miss


### PROCEEDINGS.

### National Dental Association, Omaha, Nebraska, 1898.

Reported for the DENTAL REGISTER by JENNIE M. WALKER, Bays St. Louis, Miss

[*Continued from Page* 490, *October No.*]

In the report of Section II.—Dental Education and Nomenclature—the committee suggests that in future editions of text-books, now in use, and in all new text-books, a glossary of terms employed, be inserted, one of the great needs of to-day being a comprehensive dictionary of dental terms. Section II. has decided to take the first steps in this direction, and asks the co-operation of the entire National Association, suggesting that the Chairman and Secretary of each of the ten Sections take steps to secure a list of all the important terms in their respective branches, sending the same to Section II. at as early a date as possible.

---

### Cast and Model.

Abstract of Paper read by Wm. Ernest Walker. Presented by Section II.

A great deal of confusion has surrounded the two terms—"cast" and "model," and the distinction to be drawn between the two. The suggestion that "the term 'model' serve as the title for a plaster cast, which is to be reproduced in metal; 'cast' to be applied to the plaster not so reproduced," did not prove acceptable to the Committee on Nomenclature of the American Dental Association. The committee, through its chairman, Dr. S. H. Guilford, deciding that "the practice of calling all plaster casts 'models,' appears to be more acceptable;" the word "cast" being included in the list of terms, the use of which was recommended by the committee to be discontinued. This decision, however, is not universally satisfactory. Much descriptive detail may be avoided by impressing the following simple distinction upon the mind of the student or laboratory assistant: The first step is the "impression;" this forms a matrix in which is poured the plaster, which forms a "cast." This is not a model of anything, but is ready for use in the production of a vulcanite, celluloid or cast-denture. If, however, it is desired to make a die for swaged-work the cast is *converted* into a "model," by so shaping it that it will accurately represent the die which is to follow. The successive stages in the process are—1, impression; 2, cast; 3, model; 4, sand-matrix; 5, die; 6, counter-die. This consecutive series is readily fixed in the mind, and, thenceforth, there is no confusion; each term represents a definite stage in the production of an artificial denture; a clearly defined object in the mind's eye. If a student, or laboratory-man, is told to make a "cast," he simply fills the impression in the ordinary way; but if told to make a "model," he first pours the cast, then thickens it if necessary, and trims it into proper shape to serve as a model for the metal die which is to follow. It appears, then, that we should hesitate before positively adopting the word "model," to the exclusion of the term "cast."

DISCUSSION.

Dr. C. L. Goddard was glad the paper had been read, as it covers the points he makes in his Lectures on Prosthetic Dentistry. He hopes the Association will approve the distinction made in the paper.

Dr. S. H. Guilford takes exception; he has gone over the ground very carefully. The Committee on Nomenclature of the American Dental Association had thought it wise to use one term whenever possible. The only difference between a cast and a model is a half-inch in height; is that sufficient to justify the use of an additional term? Better call both "models."

Dr. Molyneaux had for years used the two terms, as defined in the paper read, and had argued in favor of that distinction at the Asbury Park meeting of the American Dental Association, but had yielded to the recommendation of the committee.

Dr. Goddard thinks there is more difference between a cast and a model than that made by Dr. Guilford; the cast, when it is to be used as a model, has not only to be increased in height, but it must be trimmed, in order that it may drop readily from the molding-sand.

Dr. Barrett: "Cast" is the proper noun to be used as the object of the process of casting; a model is not cast; a model must be manipulated with the fingers; a cast is the result of mechanical crystallization.

The point in Nomenclature was referred back to the Section for further consideration.

### Materia Medica and Therapeutics.

Abstract of Report of Section V. By Dr. J. S. Cassidy.

The acceptance of the bacterial genesis of many oral diseases has been followed by a deluge of drugs of antiseptic quality, culminating in a tendency to ignore the very new remedies and to investigate more thoroughly the value of those longer known.

The older drugs, while they may have some objectionable qualities, are comparatively simple in their chemical and therapeutic action, and are time-tried and reliable. They are non-secret and many of them not even proprietary.

Formaldehyde, though apparently new, is simply the active principle of the earth of ant-hills, which, from time immemorial, has been used as a local remedy, in Continental Europe, in the domestic treatment of swellings, bruises, suppurating sores, abscesses, neuralgia and rheumatic pains. Formalin, while a combined disinfectant, antiseptic and germicide, has irritant qualities which make it objectionable for use in the mouth, cases of serious sloughing of the gums being reported. There is, however, no better agent for destroying infective matter on steel instruments without injuring them. For this purpose it is harmless and immediately effective, the instruments, burs, excavators, etc., after cleansing them, being dipped in full strength formalin and wiped off dry.

CATAPHORESIS.

The latest phase in cataphoresis is a presentation of the definite rules of electrolysis, enabling the operator to select the proper pole to be used in connection with the cataphoretics used in dentistry. Even non-conductors are induced by the pressure of the current to penetrate the tissues further than by mere absorption.

NITROUS OXIDE.

The action of nitrous oxide was discussed in the report of Section V. The conclusion reached by Drs. Kemp and Bush, that it exerts "a specific anesthetic influence"—not merely depriving the patient of oxygen—is considered and endorsed, but the statement that its influence is due to the retention of $C O_2$ in the arterial blood is criticised. It goes through the arterial circulation unchanged, superceding for the time, iron, the oxygen carrier of the body. $C O_2$ being developed in greater quantity than usual cannot escape freely, the reduction of the ferric oxide in the blood not being accomplished, leaving the iron unprepared to convey the $C O_2$ as ferrous carbonate to the lungs. But if either pure oxygen or air be administered simultaneously, or at short intervals, one of the physiological functions of iron is restored sufficiently to prevent cyanosis. This is simply the carbonic acid theory of anesthesia by nitrous oxide—$N_2O$ revived, and it is gratifying to know that it is being investigated by such an institution as Johns-Hopkins University.

### Anatomy, Pathology and Surgery.

Abstract of Report of Dr. W. C. Barrett. Section VII.

(The journalistic literature of the past year.)

### THE VASCULAR SUPPLY OF THE TOOTH-PULP.

The papers of Dr. J. L. Williams and his micro-photographs support the view, held by some anatomists, that the vascular supply of the tooth-pulp must come from the pericementum, and that preparations and cuts, representing the dental arteries as giving off branches which enter at the apical foramen of each tooth root, are a misrepresentation of the actual condition; the pericementum being in fact the placental organ that supplies the tooth-pulp with nutriment in all cases.

On the other hand, Dr. M. H. Cryer's preparations lead to the belief that the old text-books were right. But nothing is yet conclusively proved. Dr. Williams must show that his preparations positively bear the interpretation given them by him; and Dr. Cryer must determine, by injection, that there is a direct communication of the dental pulp with the dental artery, before his deductions can be accepted as final. The subject is one that may well engage the attention of microscopists and anatomists during the coming year.

### THE BROPHY OPERATION.

Prof. Brophy has performed his operation for the radical cure of cleft-palate a number of times during the past year, and in every case, so far as known, it has proven a success.

At a very early age—preferably within a week of birth—the separated osseous borders of the palatal arch are brought into coaptation, and held thus until union has taken place; subsequently closing the soft palate, of which the edges have been brought nearly into apposition by the previous closing of the hard palate, so that there is but little stretching of the tissue, or drawing away of the closed velum from the posterior walls of the pharynx—a fatal defect in the operation of staphylorrhaphy, resulting in failure to effect any radical improvement of speech or deglutition. Bringing together the divided palatal walls, in the "Brophy operation," necessarily reduces the maxillary arch, the upper jaw being contracted from one-third to one-half in some cases, leaving it not more than three-fourths of an inch in breath. The most surprising fact is that, if the operation is performed early enough, the superior jaw develops to normal size, the upper and lower deciduous teeth erupting in perfect occlusion.

In the discussion of this portion of the report, Dr. Patterson said that as he had formerly been opposed to the Brophy operation, he now wished to place himself on record as being in favor of it, having been converted, through his knowledge of three successful cases. He had never seen, by any other method, any cases that could be called a success in any shape or form. The only object of the operation for cleft-palate is the improvement of the articulation, and in this the Brophy operation is a success.

Dr. Barrett considers the operation a great advance in surgery. As the Great Physician anointed the eyes of the blind and they saw, so modern dental surgery touches the lips of the dumb and they speak. It is a miracle as profound as that of opening the eyes of the blind. For the first time in the history of surgery, cleft-palate can be cured. It is the grandest triumph of our profession, for it is oral surgery. The operation is radical in its nature and is performed within a week after birth, preferably—not after the first year.

Dr. Brophy has recently performed his 495th-6th-and-7th operations. In only one case did the infant die, and then not as the result of the operation.

On motion of Dr. Eug. H. Smith, Dr. Brophy was invited to prepare an address on the subject of "Surgical Operation for Cleft-Palate" for the next meeting of the Association.

He accepted the invitation, and promised, if the meeting were held within a convenient radius of Chicago, to present four or five small children who had been operated upon at a very early age, that the results of the operation might be seen.

### PYORRHEA ALVEOLARIS.

There has been nothing definitely settled, as yet, as to the various theories entertained concerning the different hypothoses advanced as to the etiology and pathology of this disease. The condition presents many phases, some of which are local and some probably general. Of these there should be a differentiation, and a clear line drawn, and the symtomotography of the various morbific changes outlined. That in all cases, not purely local in character, the pericementum is seriously involved, seems apparent, because its destruction—either by the extraction of the involved tooth, or by the temporary removal and subsequent replantation and consequent new pericemental formation—affords an almost infallible cure. The subject needs further elucidation.

In the discussion of this subject, Dr. Patterson said that he did not believe that there was such a thing as pyorrhea alveolaris without deposits. He believes they are always there, though not readily noticeable; a *shaving* process will often remove it when the probe fails to find it, and a cure follows, showing that it was there. On a tooth that has been removed a strong eye-glass will often show very small patches, not otherwise to be detected, but which are as irritating as larger accumulations.

Dr. Barrett: Where there are pockets there are always deposits. But in many cases there are no pockets, but a wasting purulent condition of the pericemental membrane.

### TREATMENT OF THE ANTRUM.

A difference of views exists as to the use of drainage tubes in the treatment of degeneration of the mucus membrane of the antral sinus. In opening the antrum the object is either to afford the means of exploration, or to give drainage to pus. If the former, there is no necessity for keeping the opening patulous. If the latter, tubes, plugs and tents tend to retain the discharge and perpetuate the septic condition. The very first principle of antiseptic surgery is that pus must at once be eliminated, at any risk, being infective and degenerative in its very nature. The presence of even food and saliva in the sinus is infinitely to be preferred to that of pus, as it will work its way downward by the way through which it was forced. Food does not rise against gravity, and saliva seeks the most dependent point. If the opening is as large as it should be, to serve for drainage, it will not be closed by hypertrophic growths, as is urged, and if they are formed they are easily removed with a bistoury, and their reformation prevented by a simple cauterant. The irritant presence of a tube, on the other hand, is certain to cause exuberant granulations.

In the discussion of this portion of the report of Section VII. Dr. Brophy said that naturally the opening must be located at the base of the cavity—the most dependent part—and must be sufficiently large to permit of thorough exploration and ocular observation. The proper point is naturally the canine fossa. The origin of the disease must be ascertained, whether it is of dental origin, alveolar abscess, etc. The nasal passages must be closely examined ; ascertain whether the frontal sinus is involved ; observe the ethmoid cells. If there is diseased bone it must all be removed, as it will be an obstacle in the process of repair. A large opening is necessary in serious cases, and a canula with a flange on the outside to hold it in place, is essential to thorough irrigation of the cavities. A small opening is of no avail except for the temporary evacuation of the cavity.

Dr. G. V. I. Brown prefers to pack the cavity with anti septic gauze, rather than use a tube, which is a source of disease. The gauze packing does not interfere with the process of healthy granulation, and does not offer the disadvantages of tubes.

Dr. Eug. H. Smith said that the first step is diagnosis. If the disease is due to dental irritation the antrum may be evacuated through the canal of the tooth which caused the trouble. Proper treatment of the dead tooth, removing the cause of the disease, may be sufficient, without any large opening.

Dr. Peirce described a case due to dental irritation, which was treated through a small opening along the buccal roots. The statement that a large opening is always necessary should be modified. If diagnosis is carefully made, a small opening will often be found to answer all purposes.

Dr. Crawford said there is no field in which more mistakes in diagnosis and in treatment are made, than in antral troubles.

He had seen no less than five cases recently, starting from the teeth and opening into the nasal cavity, diagnosed and treated as antral trouble.

Dr. Brophy said that in serious, chronic cases, a large opening is necessary. If the cause of the trouble is a simple alveolar abscess, the case is different. But in a large majority of cases, he believes the repeated washings and drenchings do more harm than good.

THE ACTION OF ARSENIC.

Dr. B. N. Smith, in a paper on arsenic for devitalizing tooth pulps, presented the following points:

The precise nature of the action of arsenic, especially in its application to the dental pulp, has never been demonstrated nor explained. That it causes death to the pulp by congestion and strangulation at the foraminal apex is shown to be an error by the fact that it is equally fatal to the pulp of a partially developed tooth with open apex, and the end completely patulous. Death, in consequence of the internal administration of arsenic, appears to be due to nervous shock, terminating in complete collapse, seeming to indicate some special dynamic energy. The minimum amount required for pulp devitalization, when directly applied, has not been definitely ascertained. A second application is a great mistake. If the tissue within the pulp chamber is found devitalized there is no question concerning that within the root canals. The sensitive point at the apex, so often encountered, is not vital pulp tissue, but is due to an intensely inflamed, irritable condition of the corpuscles of the cementum at the extremity of the root, due to the use of an unnecessarily large amount applied to the pulp, and left in the tooth too long.

A second application may destroy the vitality of the apical cementum altogether and induce serious trouble. Instead of another application of the irritant poison the contrary treatment should be inaugurated. Neutralize the arsenic by an application of dialyzed iron, and use soothing anodynes until all danger of further action of the poison has passed away. Then clean and fill the canal.

Section VII. offered three other papers—(1) Anatomy; a

Critique, by Wm. Ernest Walker; (2) The Comparative Method of Teaching Dental Anatomy, by A. H. Thompson; (3) Paper illustrated with slides, by M. H. Cryer.

Before reading his paper, Dr. Walker asked permission to test a point in anatomy, which he had made a subject of investigation; namely, whether the molars touch, when the incisors are placed edge-to-edge, in biting contact.

In response five of those present raised the hand, signifying that their molars do so touch; thirty-five signified that they do not touch.

Dr. Walker then read his paper, of which we give a brief abstract:

While congratulating the dental profession on the great improvements in the 1897 edition of Gray's Anatomy, especially in the section on the mouth and teeth, he directs attention to a few minor errors which have crept into this section, to which the attention of students should be directed. In the definitions of the surfaces of the teeth (page 932) they are named as labial, lingual, buccal, distal and proximal. The mesial surface is not named, proximal being defined as the reverse of distal, which is defined as "the surface away from the mesial line." The word *mesial* not being defined, looking it up in his dictionary the student is led to believe that the mesial line must pass antero-posteriorly between the superior incisors, parallel with the tongue, and that consequently the surface of the tooth which is "toward the mesial line"—the definition of proximal—must face the sides of the tongue. This was certainly not the idea intended to be conveyed by the definition of the proximal surface of a tooth. In the text, moreover, proximal is not used as the name of a tooth surface, except in the definition, mesial being substituted, although nowhere defined.

Mesial and distal are both used in conformity with the definitions of Dr. G. V. Black as found in the Transactions American Dental Association, 1895: "Distal—away from the median line of the face, following the curve of the dental arch;" "Mesial—toward the median line of the face, following the curve of the dental arch." Proximate (proximal) "applied to a surface of a tooth (either distal or mesial) which is next to another tooth."

These definitions are more satisfactory than those given in the 1897 edition of Gray's Anatomy.

On page 935 we read: "The movement of the human mandible is forward and downward, the resultant of these directions being an oblique line, upon an average of 35 degrees from the horizontal plane." As authority for this statement, a foot note refers to "W. E. Walker, *Dental Cosmos*, 1896."

The statement as given, however, is misleading. *Mandible* should read "mandibular condyle;" "35 degrees from the horizontal plane" should read "40 degrees from the facial line," as there is no "horizontal plane" in the head. The plane of condyle movement forms an average angle of 35 degrees with the plane of occlusion, the latter forming an average angle of 15 degrees to a plane perpendicular to the facial line—a plane which could be called *horizontal*, only when the facial line is vertical—which plane would form an average angle of 50 degrees with the plane of condyle movement, instead of 35, as stated in the text.

Dr. Walker quoted from the text the statement that, "When the lower jaw is advanced until the cutting edges of the incisors are in contact, the jaws are separated; but as the highest part of the lower arch—the third molar—advances, it meets and rests upon the second molar of the upper arch, and thus undue strain of the incisors is obviated." A similar statement is made by Bodecker, in his "Anatomy and Pathology of the Teeth," and is also repeated and amplified in Dr. Burchard's recently issued "Dental Pathology," as follows: "While the jaws are in position, with the incisors in occlusal contact, they do not bear alone the stress of whatever muscular force is applied, but it will be seen that the distal cusps of the third molars, the highest points of the dental arch, advance and meet the distal cusps of the upper second molars, so that when the incisors are in edge-to-edge occlusion, although all of the other teeth are separated to an extent governed by the overbite, the dental arch is supported posteriorly by contact of the last molars, thus preventing undue stress upon the incisors."

Dr. Walker said that his studies of the articulation and occlusion of the human jaws had convinced him that this statement is not based on fact. In order to test the point, he had, on one occasion, taken casts of students' mouths, at the dental college, taking them as they came—rejecting only those mouths in which very many teeth had been extracted—until he had taken, and was prepared to exhibit, thirty-three casts of jaws with the teeth in edge-to-edge occlusion. Of these, seven were excluded because of abnormality, there being no over-bite in the position of rest. Of the twenty-six left, twenty-two had the molars *entirely free*, when the incisors were thus placed end to end. One has contact of the molars on the right side only, where the second and third molars have come forward, due to loss of the first molar. The second case, in which there is molar contact, has lost the left upper second bicuspid, and left lower first molar; also, the right lower second bicuspid and the first molar. The third of the "contact" cases has no bicuspids and only one molar on the left lower side, and shows great wear of the incisors. He also has casts of three unusually symmetrical mouths, with the teeth both in normal occlusion, and also in the *biting* position. These casts also fully sustain the position that—as a rule—the molars are not in contact when the incisors are in edge-to-edge contact. He has also made it a practice to question intelligent individuals on this point, and finds that it is a rare exception, rather than the rule, that the molars are thus in contact. He has concluded that the idea must have originated in a misunderstanding, by Dr. Bodecker, of Dr. Bonwill's advice to construct *artificial* dentures with what he calls "a balancing articulation;" making the artificial molars touch at the same time that the incisors meet, in order to support *the plate*, and prevent its displacement in mastication. The error, which appears to have thus originated with Dr. Bodecker, has apparently been perpetuated by subsequent writers, without any effort being made to either confirm or refute it, as far as is known to the writer, who requests that if investigations have been made on this point, the results be made known, that a definite conclusion may be reached as to the correctness, or otherwise, of the statement with which he takes issue.

### The Comparative Method of Teaching Dental Anatomy.

Abstract of Paper by A. H. Thompson, Topeka, Kansas; offered by Section VII.

Dr. Thompson spoke of the wonderful collection of skulls and teeth exhibited at Old Point Comfort, and the papers read, as proof of the growing interest in the subject of comparative Odontography, and a matter of congratulation to those who have labored for years to impress upon the faculties of our colleges its value as a contributory science; and the desirability of its recognition in our curriculum as throwing light upon human odontology.

Dental anatomy was long taught by study of the human teeth alone. The limited human denture comprised the whole range of the subject of odontography as taught in our books and in our schools. The teeth of the lower animals were regarded as mere curiosities. The teeth, and in fact all the organs of the human body, were studied as if man were a special creation—independent of all other forms of life. But all this has been changed, and methods of study have been revolutionized.

All life is now regarded as a unit—man is but a part, a very insignificant part, of the realm of nature. He was not a special creation; he has no kingdom of his own; he is a vertebrate like other vertebrates; no class of his own, he is a mammal like other mammals; no order of his own, he is a primate sharing this distinction with apes and monkeys. The skeleton of the higher apes resembles that of man more than it does that of the monkey below them. This is also true of the molar teeth, and it is proper and rational that the organizations of the lower animals should be studied in connection with that of man, for the knowledge to be gained by comparison of related types of structure. This is the only truly scientific method. The paths of evolution have been marked out and the life-history of the lower animals is now well understood.

The phylogeny, or history of the evolution of the type of man from the lowest forms, has been made possible only by the comparative method. Of his ontogeny—the development of the individual in the embryo—we have learned much by comparing the various stages of growth with similar stages of the development of lower animals. The human embryo recalls types of lower forms, at various stages of its development. This is also true of the various organs, of which much is learned through the study of their evolution in the lower animals, from the lowest organisms in which a suggestion of these organs may be found, to the highest types.

The comparative method having been thus applied to the study of other organs, it is but rational that it should be similarly employed in the study of the teeth of man.

The life-history of the teeth is of special interest to us as dentists, and we must consider it from the scientific standpoint.

The teeth were developed for a functional purpose—that is, the reduction of food preparatory to digestion. By the investigation of the teeth of the lower animals, we learn much concerning the origin and development of these organs. We learn that, morphologically, they are mere dermal structures and appendages, modified and elaborated for food-reducing purposes. The variety of jaw movements is important in the influence that this force has upon the form and size of the jaws and the masticating apparatus, and the tremendous effect it has upon the forms and positions of the teeth.

The adaptation of tooth forms to the various kinds of food is most wonderful and beautiful, and the various types illustrate the variations in the forms of the teeth, as adapted to their varied functions; the incisors for cutting purposes; the canines as prehensile organs, of extraordinary development and form in some of the lower animals, but greatly reduced in man; the molars as crushing and masticating organs of greatly varying forms, from simple to complex, adapted to various forms of food. All this offers a most fascinating study taking us into fields where the highest genius of our race has exercised its powers, and where great biological problems bearing upon the origin of man have received illumination. The evolution of the jaws and of jaw-movements, and the principles of occlusion, a thorough knowledge of which is of such vital importance to us, in our every-day operations, is greatly in need of illumination. We have studied only the jaws of man, which are but rudimentary, as compared with the jaws of many other animals. We need to go far afield, to learn more of the mechanism of the jaws from animals in whom it is more highly specialized. The relation of tooth-forms to jaw-movement is a most interesting branch of the subject, there being an exact relationship between tooth-forms and their functions and jaw-movements. It is a field that offers great possibilities of discovery for the student.

In the discussion of this paper, Dr. Barrett said he did not agree with the essayist that the mastication of food was the primary function of the teeth. Man, in his *species* is the primal object; in his *class*, mammalia, he sinks into insignificance. The teeth are primarily weapons of offense and defense; secondarily, they are organs of prehension; *perhaps*, thirdly, they are organs of mastication. In the study of comparative anatomy, we must rise to a higher plane than that of our daily labor; we must study from the scientific, not from the standpoint of the bread-and-butter question. It is not true science when we talk of dentistry; it is applied science, but it is not pure science. The teeth are not, first of all, organs of mastication.

Dr. Crawford said that he was greatly interested in the development of this question. With all due deference for Dr. Barrett, he did not believe that the human teeth were primarily organs of offense and defense. He thought these questions should be studied from the standpoint of nature, and further than that, in their bearings upon life, character and civilization.

Dr. Thompson, in closing the discussion, said that he had not felt under obligation to consider the primary functions of teeth, but he does believe that their primary function is food-reduction, for the nutrition of the system. Their function as weapons or tools is secondary, not primary. He referred to Dr. Walker's studies of the teeth and jaw-movements, upon which, he said, Dr. Walker had thrown a great deal of light, and he hoped he would do more work in this direction. We can learn much by the study of the lower animals.

There being no lantern available, for the exhibition of Dr. Cryer's slides, the remaining paper offered by Section VII. was not read.

---

Section VIII. offered no report.

Section IX. offered one paper, by Dr. C. S. Case, entitled, "Answer to the Criticisms of My Paper entitled, 'Principles of Force and Anchorage in the Movement of Teeth.'"

Dr. Case read this paper, of which we give an outline.

He said that had he anticipated at the last meeting of the American Dental Association—a meeting of representative American dentists—that a principle, founded upon the most simple and well-established laws of physics, would be criticised as of no practical or scientific value whatever, he would have been prepared, as on the present occasion, with apparatus to practically demonstrate its truth, and also with models, which speak for themselves of its value, and which fairly represent hundreds of similar cases in his practice.

As it was, with nothing to demonstrate the practicability of his claim, the discussion had resulted in a controversy of opinion alone, which left the profession at large divided in opinion as to the practical value of his methods in general.

The question which occasioned the principal contention was in regard to the action of force, when applied to a rigid bar or extension above the gingival margin of the crown. Dr. Case's methods are based upon the principle that when force is applied anywhere upon a bar or extension above and on the outside of the gum, made rigid with the crown, it will have exactly the same effect as if applied to the root itself, at a point on a line with the direction of said force.

To place this claim beyond controversy, Dr. Case had on exhibition an apparatus consisting of a large wooden tooth suspended in an upright position by being attached along the posterior surface of its root to five steel spiral springs, which in turn were fastened to and supported by a frame. These springs were so adjusted that a given force applied to each, separately, would produce the same amount of motion in compression or extension.

Force applied to this tooth, in a manner similar to that which is possible with a dental regulating apparatus, represents the direction, and approximately the energy, expended at different locations upon the root, the movement being registered by hands moving on dials in the same direction that the portion of the root moves. To the front of the tooth was attached an upright extension, similar to that extending above the gum margin; in Dr. Case's regulating apparatus the extension having notches at different points for the attachment of power bars.

Power applied at the highest point upon the crown, possible with an ordinary appliance, showed the tooth tipped from an upright position, the cervical portion moving in the direction of the applied power, the apical portion moving about one-fifth as far in the opposite direction. Power applied above this point soon found a place where the end of the root ceased to move in the opposite direction, but remained stationary, as a pivotal point to the movement in other parts. Power applied upon the upright bar, at a point whose line of force intersects the root in the center of its area of resistance, moved the whole tooth equally, in an upright position, in the direction of the force.

The apparatus thus shows that force acting upon a bar made rigid with the crown, and extending in front of the gum, would produce the same effect as if applied to the root itself, and the same as if the space between the bar and root were one solid piece—as shown in the apparatus by filling the space with a piece of wood cut to exactly fill the space—the result being the same, whether the space was filled in with the block, or left vacant.

In a regulating apparatus constructed upon this principle allowance must, of course, be made for the varying stability of the surroundings of the natural tooth, and the influence of the variable size, shape and number of the roots; but the principle was demonstrated, showing the direction and amount of force that the root can be made to exert upon its surroundings.

The practical application of the principles of dynamics, so far as we can utilize them, is the only scientific course to be pursued in the practice and teaching of orthodontia.

Dr. Case next demonstrated, by means of a box of modeling clay and a rod, the direction of the force exerted by different portions of the root of a tooth, as illustrated by a post driven one-half its length into clayey soil. This was described and illustrated, but not demonstrated at the preceding meeting. A mathematically correct demonstration would demand a medium absolutely and unchangeably uniform throughout in elasticity and resistance, a medium purely hypothetical and especially dissimilar to a tooth situated in the alveolar process; but the principle is important and valuable, *viz:* that when power is applied at different localities upon the exposed portion of an incisor tooth, the relation of force exerted at different portions of the imbedded end is practically maintained. If it is desired to protrude the end of the root of an anterior tooth to give a fuller contour to the lip, power should be applied at the extreme incisal end of the crown. If, on the other hand, it is desired to retrude the tooth, with no forward tipping of the root, force should be applied at the gingival border. An upright bar made rigid with the crown and extending above the gingival border affords opportunity for still other directions of force.

To the objection raised to the employment of bands encircling the teeth, necessitating separations for their adjustment, Dr. Case said that it was rarely necessary to separate more than could be accomplished by leaving a piece of waxed tape between the teeth over night. His bands are prepared with the greatest care and accuracy, rolled to exact sizes, ranging from three to ten thousandths of an inch in thickness, and of different widths. As five thousandths of an inch, or No. 36 B. & S. gauge, is the thickness of ordinary ledger paper, while the bands principally used are three and three and one-half thousandths of an inch thick, the separations necessary are a matter of very little moment, as they soon close after the apparatus, or retainer, is removed.

In the discussion of this paper, Dr. H. A. Smith spoke of a case recently treated by Dr. Case, which had been referred to Dr. Smith, after treatment by three other dentists. The trouble was really with the inferior maxillary, while all the work attempted had been put upon the superior. When Dr. Smith saw the case he said, "There is but one man who can manage this case, and that is Dr. Case." Months had been wasted in labor that was utterly objectionable. There had been no study of contour. Such cases should be referred to a specialist, a master in the art and science of orthodontia.

Dr. John S. Marshall said that when Dr. Case had presented the subject to him he had thought he was wrong on the question of leverage, but his exposition of the subject to-day had made a convert of him, and he desired to give his testimony to that effect.

Dr. S. H. Guilford said that Dr. Case had undoubtedly accomplished fine results for his patients, but that in the matter of the principles of physics he is not correct. Dr. Guilford then criticised a number of Dr. Case's illustrations and the application made of them; also, that many of the terms which Dr. Case uses are absolutely unscientific, citing as examples "fulcrum area," "static anchorage," "mechanical fulcrum," etc. He also criticised the use of a rod moving in modeling clay, to illustrate the movement of a tooth in the alveolar process with its tissues of varying density and power of resistance.

Dr. Case replied that he was glad to have a man of Dr. Guilford's recognized ability point out his errors, but that he deprecated the criticism of illustrations used last year, but not referred to in the present paper. He had no objection to the substitution of "stationary" for "static," if the latter is not the correct term.

Section I. was recalled, and Dr. V. H. Jackson read his paper, entitled, "The V. H. Jackson Compensating Bridge."

The bridge thus designated is designed to fill the space between teeth having vital pulps and unbroken crowns, but of such shape, or inclination, that the space between the tops of the crowns is less than that at the necks, and where it is desirable, not to cut away the enamel to get parallel piers. Dr. Jackson considers it "inexcusably bad practice" to cut away the enamel from teeth that have vitality, in cases where the desired result can be obtained by methods less destructive. In the present method the bridge is made either with a saddle resting upon the gum, or is suspended from the abutment teeth by its attachments. The bridge is introduced from either the lingual or the buccal side of the arch, as determined by the angle of the teeth and the contour of the gum. An accurate impression, and a very accurate plaster model of the teeth that are to serve as abutments, and of the contour of the gum between them, are essential. A collar is adjusted to each of the anchorage-teeth of the model, broad enough generally to reach from the gum to the grinding surface, leaving one end of the collar about one-eighth of an inch longer than the circumference of the tooth; the end to be bent outward, at a right angle, and strengthened with an additional layer of gold, and have a hole passing through it for the reception of a bolt. The collar must be well contoured to fit the surface of the tooth. The abutment tooth must, if necessary, be wedged away sufficiently to allow the collar to pass freely before the impression is taken. Several methods of making the collar tight around the tooth have been utilized; for most cases, the tap-bolt and nut gives the best satisfaction, made of hard, non-corrosive metal and provided with a deep, strong thread, having the thickness of the nut about one-half the labial diameter of the tooth. In some cases funnel-shaped tubes are used to receive conical nuts. The bolt may enter from either the buccal or the lingual side. A strong clip of plate metal is extended from the collar on to the grinding surface of the tooth to prevent the collar from slipping toward the gum. The clip should be strong enough to withstand any pressure on the bridge that may be made in mastication. If the bridge is to rest in contact with the gum, a saddle of thin platinum, or gold, is swaged to fit the portion of the gum that is to be covered. When the collars and saddle have been accurately fitted to the model, porcelain teeth are backed, articulated, and waxed into place, and the parts united as in ordinary bridge-work; or the collars and saddle having been adjusted, plain rubber teeth may be used and the case packed with rubber and vulcanized, as in making a partial rubber plate. In suitable cases the Mason detachable porcelains may be used. For crossing the labial side of a cuspid a loop of gold wire mars appearance less than a collar. This should have the inner side flattened; one end of the wire may be threaded and made to project through the tube a little and engage with the nut; the other end bent at a right angle, to form a hook to catch in a hole in the distal side of the cap, which should cover the mesial, distal, and lingual sides of the cuspid. Before setting the bridge, the teeth for anchorage are polished and made dry, the inner sides of the collars covered with a thick solution of chlora-percha; the collar-bands are sprung backward to pass over the teeth as the bridge is adjusted. The collars are then drawn around the teeth and the tap-bolts screwed quickly to place. After the bridge has been made firm, the small openings around the heads of the screws may be filled with gutta-percha; in some cases a bolt with roughened surface can be retained with oxyphosphate cement, and not depend alone upon the screw thread.

A large number of cases illustrating the various applications of this method were described in detail, and models of cases, with bridges in position, were passed around for examination.

The paper was passed without discussion, the hour for final adjournment having arrived.

After the usual ceremonies, announcement of committees by the newly-elected President, etc., the Association adjourned to meet at Niagara Falls, the first Tuesday in August, 1899.

